# Current Trends for Lavender (*Lavandula angustifolia* Mill.) Crops and Products with Emphasis on Essential Oil Quality

**DOI:** 10.3390/plants12020357

**Published:** 2023-01-12

**Authors:** Ioana Crișan, Andreea Ona, Dan Vârban, Leon Muntean, Rodica Vârban, Andrei Stoie, Tania Mihăiescu, Adriana Morea

**Affiliations:** 1 Department of Botany, Faculty of Agriculture, University of Agricultural Sciences and Veterinary Medicine of Cluj-Napoca, Calea Mănăștur Street No. 3-5, 400372 Cluj-Napoca, Romania; 2 Department of Genetics and Plant Breeding, Faculty of Agriculture, University of Agricultural Sciences and Veterinary Medicine of Cluj-Napoca, Calea Mănăștur Street No. 3-5, 400372 Cluj-Napoca, Romania; 3 Department of Crop Technologies, Faculty of Agriculture, University of Agricultural Sciences and Veterinary Medicine of Cluj-Napoca, Calea Mănăștur Street No. 3-5, 400372 Cluj-Napoca, Romania; 4 Department of Engineering and Environmental Protection, Faculty of Agriculture, University of Agricultural Sciences and Veterinary Medicine of Cluj-Napoca, Calea Mănăștur Street No. 3-5, 400372 Cluj-Napoca, Romania; 5 Department of Agritourism and Processing of Agricultural Products, Faculty of Agriculture, University of Agricultural Sciences and Veterinary Medicine of Cluj-Napoca, Calea Mănăștur Street No. 3-5, 400372 Cluj-Napoca, Romania

**Keywords:** properties, composition, volatiles, standards, farm, commercial

## Abstract

Lavender is in the research spotlight due to its increasing economic importance, while market demand is expected to continue to grow. Among the hundreds of essential-oil-bearing plants, *Lavandula angustifolia* Mill. remains one of the most valuable. This paper explores the lavender chain timeline from crop to products, examining the expanding knowledge on the characteristics, phytochemical profile and functional potential of lavender that could lead to new products and uses. Lavender crops can be expanded without competing for productive land, instead using marginal, contaminated or unproductive land. A novel cultivation trend proposes leveraging agri-background biodiversity, arbuscular mycorrhiza and the natural enemies of pests for healthy crops. Together with breeding efforts targeting highly performant genotypes with complex volatile profiles coupled with resistance to specific biotic (particularly *Phytoplasma*) and abiotic (salt, heavy metals) stressors, industry could have a steady supply of high-quality raw material. Besides the expansion of the uses of essential oil in cosmetics, pharmaceuticals, food and environmental and agri-applications, novel channels have appeared for the use of the solid by-product, which is rich in polyphenols and polysaccharides; these channels have the potential to create additional streams of value. The stabilization and optimization of techno-functional delivery systems through the encapsulation of essential oil can extend shelf-life and enhance biological activity efficiency.

## 1. Introduction 

The market for medicinal and aromatic plants (MAPs) is growing because of the increasing demand for specialty materials derived from them that have expanding and diverse uses [1]. Out of all the specialty materials derived from MAPs, the essential oils are some of the most valuable. It is estimated that around 3000 essential oils are known today, but only around 150 have commercial importance. It is difficult to know the production worldwide, but available data suggest that essential oil crops worldwide cover more than half of million hectares. The European market is experiencing an increasing trend in production, because leading fragrance and flavoring manufacturers are located here. Under these conditions, many European countries have increased their production [2]. Lavender is one of the most valuable plants from the range of MAPs with commercial importance, and it is cultivated for industrial purposes. The essential oil extracted from lavender is the most sought-after product obtained from lavender crop because it is further incorporated in a broad range of products [3]. From the species and hybrids of the genus *Lavandula*, the essential oils obtained account for about 1500 tons annually [4]. Out of this, the worldwide production of true lavender (*Lavandula angustifolia* Mill.) essential oil is estimated at 300–500 tons per year [5]. The quantity of lavender essential oil found in trade each year is difficult to assess, given that official figures do not account for the amount of adulteration or for synthetic lavender essential oil on the market [6]. France was among the original countries that cultivated lavender, and among the first exporters of lavender essential oil for the worldwide cosmetic industry [7]. The leading lavender production countries are Bulgaria and France, followed by other countries such as Russia, Ukraine, Moldova, Romania, Hungary, Poland, Italy, Spain, Turkey, Morocco, United Kingdom, United States, Australia, South Africa and China [8]. Lavender plantations, both inside and outside Europe, have increased in number recently [9]. The increasing demand over the last few years has been fueled by consumer behavior. MAPs improve life quality and well-being and offer a positive experience associated with their health-promoting properties [10]. A survey in Romania showed that knowledgeable consumers of MAPs in general exhibit a preference for naturally derived products that have ecological certification [11], reflecting increasing health and environmental awareness. 

Although the cultivation of lavender has become a tradition in many parts of Europe and worldwide [7], the recent literature lacks a general overview over the entire production chain of lavender from crop to products. Both opportunities and challenges have arisen recently that affect growers and the processing sector alike [3]. 

Traditionally, the trajectory of lavender from crop to product was simple. The crop was harvested and send for distillation. The farmer profited from selling the flowering tops, and the processer from selling only the essential oil obtained therefrom, according to its quality; sometimes one entity was involved in both activities. However, this functioned on a single line of operation and dealt with a single raw material, leading to the obtainment of a single product—the essential oil, which had various further destinations of use. The issue with this approach is that it misses modern opportunities for creating additional streams of value and income. In order to maximize the beneficial outcomes, both lavender growers and the processing sector must consider complementary and secondary utilization options to make full use of the market opportunities. Literature abounds in various interesting findings, but little is yet known about when these could become implementable, or how they might connect with current consumer trends. For this reason, this review summarizes the current state of knowledge by bridging long-known cultivation practices and traditional uses with novel ones. Challenges related to emerging pests, cultivation technologies and perspectives on quality standards, as well as the factors influencing them, have been researched. 

The aim of this study was to provide an overview on the latest trends for lavender crops and products, with emphasis on essential oil quality, since essential oil is economically the most important product obtained from lavender at this time. Two objectives were defined: (1) reviewing the state of the art in lavender crops and products, with emphasis on emerging trends; (2) identifying the gaps in knowledge regarding the optimization of lavender crops and products and the increasing of opportunities. 

## 2. Origin, Botany and Phytochemistry of the Genus *Lavandula*

**Figure 1:**
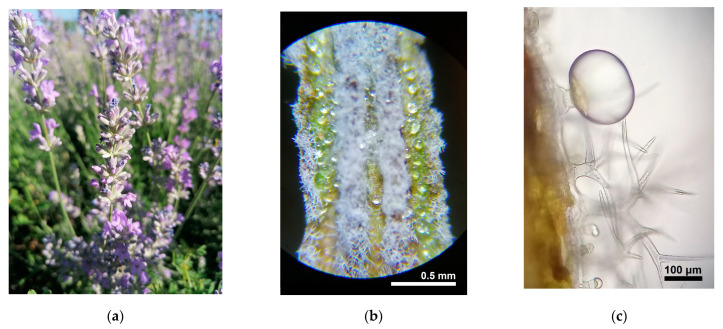
Aspects of *Lavandula angustifolia* ‘Sevtopolis’, one of the frequently cultivated genotypes in Eastern Europe: (**a**) inflorescence; (**b**) glandular trichomes located in the calyx grooves and branched trichomes with protective role, magnified 160×; (**c**) microscopic sideview of a glandular trichome and branched protective trichomes in the background, magnified 400× (original photos by Ioana C., 2022).

The genus *Lavandula* belongs to the family Lamiaceae. This is an economically important botanic family that comprises many herbs and shrubs known for their medicinal or culinary use and cultivated worldwide [12,13,14,15]. The flowers are hermaphrodite, with characteristic bi-labiate morphology and nectaries that attract bees. The plants present glandular trichomes over the aerial organs [13,16], and these can havegreater abundance on certain plant parts, depending on the species (Figure 1). These epidermal structures contain aromatic volatile oils [13]. The morphology, localization and density of these structures are distinctive features among the species of this botanic family [17]. Their density, frequency and size influence the essential oil obtained [18]. Characteristic to this family are also different glycosides of taxonomic significance [6].

The genus *Lavandula* comprises 39 species, 79 infraspecific taxa and a number of hybrids classified into 3 subgenera and 8 sections [19]. There are also about 400 registered cultivars [20]. The genus occurs naturally from the North Atlantic islands to India [19]. Within the genus, the -species most commonly cultivated are *L*. *angustifolia* (lavender or true lavender), *L*. *latifolia* Medik. (spike lavender), *L*. *stoechas* L. (Spanish lavender) and *L*. × *intermedia* Emeric ex Loisel. (lavandin). The latter is a sterile hybrid obtained from the crossing of *L. angustifolia* × *L. latifolia* [21]. For essential oil production, the most cultivated genotypes are *L. angustifolia* and *L.* × *intermedia* [7,22], but lavender oil (of *L*. *angustifolia*) is marketed at a price around 3–5 times higher than lavandin oil, as it is considered of higher quality [23]. The plants of the genus *Lavandula* are rich in phenolic compounds, with 8 anthocyanins and 19 flavones identified, and the essential composition varies across the species of the genus [6], with more than 300 terpenes (mono- and sesquiterpenes) accounted for [24]. 

*L. angustifolia,* also known as lavender, is native to the mountainous regions of the Mediterranean from Spain to France and Italy, where it grows generally at altitudes over 1500 m. There are two subspecies: ssp. *angustifolia,* native to the French and Italian Alps, and ssp. *pyrenaica,* from the Pyrenees [19]. However, some notable differences have been reported between the essential oil composition of these subspecies, with wild ssp. *pyrenaica* considered unacceptable for normal commercial grade lavender oil [6], and ssp. *angustifolia* being the most economically important out of the two - [25]. The mountain origin of this species makes it - hardy in cultivation. It grows as a shrub, reaching 50 cm in width, flowering from the middle of June to July [7]. This species is distinguished by 3–9 flowered cymes, nutlets with a basal scar, bilobed stigma and a corolla tube twice the length of the calyx. The leaves are simple and entire [7,19]. Like many members of Lamiaceae family, plants of this species also present capitate and peltate trichomes on the aerial organs [26], but in lavender, these reach highest abundance on flower calyx (Figure 1) [26,27,28]. The trichome density can vary to a certain degree between cultivars and due to nutritive supply [29]. The constituents of essential oils from aromatic plants are considered secondary metabolites [17], and have various roles in the life of plant, acting as attractive agents for pollinators, signaling between plants and intervening in some defense mechanisms against biotic and abiotic stressors [22]. The main components of the essential oils of the commercial genotypes of lavender are linalool and linalyl acetate (in amounts exceeding 20% each) [6]. 

## 3. Cultivation Challenges and Trends for *L. angustifolia* Crop

### 3.1. Cultivated Plant Habit 

As for the plant’s life-form, in temperate conditions of Europe, lavender behaves as chamaephyte, according to the Raunkiaer classification system. The perennating buds are located on persistent shoots above the soil surface [30]. Lavender is botanically classified as a perennial, evergreen subshrub [7,31], but with pruning can be trained into a more herbaceous habit, although the plant still forms some woody stems and twigs [32]. This plant presents a lignified root able to reach around 2 m or more into the soil in cultivation. The stem ramifies directly from the base to form a bush of 30 to 70 cm in height, that is maintained in a globular form by regular pruning and tops trimming at harvest. The woody stems have flaky bark. The inflorescence forms on the upper part of the plant, with one inflorescence per flowering stem. The inflorescence presents a variable number of pseudo-verticillasters (3–8) each with 2–7 florets [5]. The horticultural classification distinguishes three categories of *L. angustifolia*: dwarf cultivars, medium-sized and giant cultivars according to the growth habit. The level of compactness also varies [33]. 

### 3.2. Requirements for Environmental Factors

Lavender prefers a climate with warm summer and cool winter [34]. However, due to its high ecological plasticity, this species can be grown in a wide range of geographical regions. It requires a relatively high temperature for germination and for entering into the vegetation stage: around 10–15 °C [5]. Young plants are more sensitive to extreme weather events, but mature plants are hardy. Regions with consistently wet summers can cause fungal infection in plants, and are less suitable for crops [34]. Lavender plants can make use of less fertile soils, eroded soils and steep land [5], but prefers crumbly soil that is moderately fertile with neutral (6.4) to slightly alkaline pH (8.2) reaction. Poorly drained and highly acidic soils are less suitable. Water requirements are moderate, and the plants are resistant to occasional drought, but persistent long periods of dryness can wilt the plants. Young plants require moisture in the soil during their first year [34]. In conditions from Poland, a study showed that the use of supplementary irrigation in growing lavender caused an increase in hydrogenated compounds in the essential oil, while in non-irrigated plants, the oxidized compounds were higher compared to irrigated plants [35]. Regarding drought-stress mitigation, an experiment showed that spraying abscisic acid could have positive effects [36], and should be further tested in field conditions, due to the possibility of its use as an intervention measure in case of drought. 

### 3.3. Crop Establishment and Propagation

#### 3.3.1. Propagation

Lavender plants can be propagated by seed, cuttings, plant division, layering or tissue culture [34,37]. The most common and widely used method is cuttings. This method is used for propagating lavender for both nursery stock and field cropping [34].This method is used to obtain clonal lavender plants corresponding to the standards required for crop plants that provide the biological material used for essential oil extraction [38].

Cuttings are taken in autumn or in spring (September, October, March or April) from healthy plants 3–5 years old [31]. Cuttings can be of two types [34,39]: (1) tip cuttings 3–8 cm long from the green growth of mature plants, or (2) cuttings taken with a heel with wood from a previous season. Cuttings should have the leaves removed at the basal part and the cut area disinfected by soaking in hypochlorite or some other solution, and additionally, before inserting in moist rooting mix, they might also be treated with hormone (Indole-3-butyric acid 2000 ppm). Cuttings can be kept for rooting in a cold frame or unheated greenhouse but should be protected from frost and direct sunlight, with regular misting. Rooted plants should be obtained in a few weeks, depending on temperature. The tips are normally pinched when transplanting to encourage branching. Planting in the field can take place after 6–8 weeks and during adequate weather conditions [34]. Rooting media for cuttings have proven important in regard to the rooting duration and root system architecture [40].

Propagation with seeds is not as important for commercial crops, but can be also employed in some cases [34], such as in breeding programs. This method is also used to propagate wild genotypes of lavender plants from spontaneous lavender growing in France, which can be used to obtain essential oil in accordance with the standards [38]. The thousand-seed mass is 0.9–1 g. To establish a 1 ha lavender crop, seedlings can be obtained on a surface of 100–150 m^2^ using 0.3 kg of seeds sown in nursery beds [5]. The germination is not uniform and takes longer to obtain plants suitable for establishing a field crop [31]. 

Propagation by tissue culture is also possible [41,42], but is most justified when a small quantity of cuttings is available or very large numbers of plantlets are needed [34]. An optimized in vitro regeneration protocol showed that Murashige and Skoog medium supplemented with 1.0 mg/L Benzylaminopurine (BAP) and 0.1 mg/L Naphthaleneacetic acid (NAA) was optimum for the highest number of shoots obtained in this species by direct organogenesis; the plantlets obtained by this method further adapted ex vitro with a high survival rate (>80%) [42]. However, the plants multiplicated in vitro must also be verified further in field conditions in regard to the quality of the essential oil obtained. It has been proposed that biotechnological studies as well as agronomical field comparisons shall continue to be researched [41]. 

Layering is another vegetative propagation method, when branches close to the ground are either covered with soil [34], or small mounds are created to come in contact with lower branches [31]. Once these sections are rooted, they can be cut and replanted [34]. This method, however, is not ideal for the propagation of commercial-scale lavender crops, but could be used to obtain clonal plants to replace missing plants in the rows in the field. 

#### 3.3.2. Field Crops 

Lavender is a perennial crop that can be cultivated on the same spot for 8–15 years [31] or for 20–30 years [5]. The longevity of the crop depends on adequate pruning [31]. The crop can be started anytime during spring until autumn, but farmers now know that planting in autumn produces more flowers in the next year. At the time of crop establishment, it is important to ensure adequate soil preparation [31]. Commercial plantations are usually established on gently to moderately sloping land [34] with a slope angle no greater than 15° [5], to ensure good drainage and to avoid waterlogging. Soil ameliorants can be used to improve soil conditions; for instance, gypsum can be used for heavy sodic clay soil to help break clods. For clay soils or sandy soils, the addition of organic matter can improve texture. Mulching with gravel or wood chips or other organic types might also be beneficial in some cases. Spoon drains can be created to channel water run-off. Sand slitting can be adopted as a simpler method, and involves narrow slits in the soil filled with sand to rapidly drain excess water. Subsurface drains might be required to fix waterlogging if the terrain has a persistent issue [34]. 

Seedlings and rooted cuttings can be planted in autumn during the months of September and October, with 100 cm between rows and 50–75 cm between the plants in each row, reaching a density of 13,000–20,000 plants/ha, with higher planting density on poorer soil and lower density on richer soils [5]. 

#### 3.3.3. Potted Plants and Unconventional Crops 

A study on substrates for pot cultivation of lavender showed that the use of peat and compost leads to plants with superior characteristics from an ornamental–horticulture perspective, but volatile organic compounds profile did not change across the tested substrates. The authors suggested that locally sourced materials, such as demolition aggregates, could be added to growth substrate [43]. Due to concerns related to the unsustainable exploitation of peatlands for nursery substrates and potted plants, a study showed that peat-based substrates containing 25% coniferous wood biochar could be used for lavender growth in containers without a negative influence on plant growth and quality [44]. 

The possibility of growing lavender under hydroponic and aeroponic systems was explored with promising results [45]. A study conducted in hydroponic conditions confirmed the importance of macronutrient supply in the soilless cultivation of lavender. Phosphorus (P) levels were most consequential for plant growth, while low Nitrogen (N) levels reduced the chlorophyll content of plants grown in soilless conditions [46]. Potassium (K) level influenced root development [47]. In hydroponic conditions, some compounds of essential oil obtained from leaves of lavender (borneol, camphor, 1.8-cineole, α-terpineol, myrtenal) were shown to be influenced by N, P [46] and K treatments [47]. 

Although there are some advantages in the protected cultivation of lavender, to date this approach does not have the highest economic relevance, due to some feasibility issues in upscaling crops. 

### 3.4. Crop Care

#### 3.4.1. Weed Control a Challenge for Lavender Crops 

Weed control is important and relatively difficult in lavender crops. Weeds compete with lavender plants for nutrients and negatively affect their growth. Because this is a perennial crop, in time the plants extend and cover the soil, making mechanical weed control almost impossible. Weed control by cultivator might not be most optimal method due to the risk of damaging the plants [34]. Manual hoeing can be performed between rows [48]. Coarse sand could be applied around the plants, but in hot sun it raises the temperature around the plants. Plantations sometimes make use of weed mat or plastic film sheeting; however, these cause a decrease in the activity of the beneficial soil edaphic community [34]. 

Herbicides are not routinely used, since they can wilt lavender plants when applied, and the literature lacks sufficient studies on chemical weed control in perennial aromatic plants such as lavender. An experiment conducted in Cyprus tested seven chemical herbicides (Aclonifen 2.75–3 kg/ha, Chloridazon 2–3 kg/ha, Dimethyl tetrachloroterephthalate 7.5–9 kg/ha, Flurochloridone 0.5–0.75 kg/ha, Linuron 0.7–0.8 kg/ha, Oxadiazon 0.75–1 kg/ha, Oxyfluorfen 0.5–0.75 kg/ha) for weeds in lavender crops over three years, and crop plants were inspected for symptoms of phytotoxicity several weeks later. The dry weight of weeds decreased significantly compared to the control for all the tested herbicides, indicating their effectiveness, but phytotoxicity symptoms were observed. Aclonifen caused leaf chlorosis in lavender, and plants were stunted for several weeks following treatment, suggesting that the crop could fail, but the plants eventually survived and the decrease in yield was not significant. Flurochloridone produced moderate to severe vein bleaching, causing a greater decrease in yield than Oxadiazon, Oxyfluorfen and Dimethyl tetrachloroterephthalate. Linuron caused growth inhibition in lavender plants and more severe consequences than in all the other herbicides regarding decrease in yield. Interestingly, some herbicides increased the essential oil content of lavender plants, but not significantly compared to the control [49]. 

There is emerging evidence for the use of bio-herbicides, although the concept itself is not entirely new. The basic principle is to make use of phytotoxic chemicals from certain plants, microorganisms and arthropods to inhibit the growth of target weed plants [50,51]. Allelopathic mechanisms could be used to target the most dominant weed species from the lavender crop in a given area. For example, a study conducted in the Romania—Transylvania region on a lavender crop indicated the weeds with highest occurrence were: *Echinochloa crus-galli* (L.) P. Beauv. and *Sonchus arvensis* L. [48]. According to literature, *Carum carvi* L. bioherbicide preparation could be used against *Echinochloa crus-galli*. There are more bioherbicide preparations that could be used against the common weeds among lavender crops: *Brassica napus* L. against *Convolvulus arvensis* L., *Sinapis alba* L. against *Setaria viridis* (L.) P. Beauv., the fungus *Ascochyta agropyrina* (Fairm.) Trotter against *Chenopodium album* L. and *Cirsium arvense* (L.) Scop. and *Sonchus oleraceus* L.; *Diaporthe gulyae* R.G. Shivas, S.M. Thomps. & A.J. Young against *Urtica dioica* L., to name a few [51]. With increased knowledge and advances in the field of bio-herbicides, optimized solutions for weed control could be found that are also more acceptable to the consumer of aromatic and medicinal plants. However, more studies are required to identify their effects on the specific crop plants, in order to adjust doses and select those bioherbicides that do not exert a significant negative effect on the agronomic parameters of the crop.

A wise approach is to have a preventive mindset directed towards reducing potential weed infestation of the crop. Accordingly, by maintaining the crop free of weeds through constant weeding before the weeds reach fruiting stage, the weed seed reserve from soil is slowly reduced over time, which is highly effective for eradicating annual species. A study conducted in the Romania—Transylvania region in a three-year old lavender crop reported 34 species of weeds, with a predominance of annual species [48]. Therefore, this approach might be suitable, at least in this region. The perennial weeds should be cut beneath the collet, or preferably plucked out when soil is moist. Secondly, the grassy areas adjacent to the crop should be mowed regularly to prevent weeds from reaching their fruiting stage. As an example, common Asteraceae weeds produce a large number of seeds per season that can be disseminated easily by wind due to their pappus causing quick weed invasion of the crop. Another preventive measure would be careful consideration of the location where the crop is about to be established. Natural barriers, such as forested patches nearby, or cultivated lands that are well-maintained and free of weeds, are good neighboring areas for lavender crops. 

#### 3.4.2. Pests and Diseases of Lavender Crops 

The diseases and pests of lavender crops have in the past not been regarded as a matter of great concern; but have been occasionally reported on, particularly in association with unfavorable ecological factors for crops [34]. Historically, at the end of the XX^th^ century, the *Phomopsis lavandulae* (Gabotto) Cif. & Vegni epidemic decimated lavender plantations in France. Since then, losses have been reported due to various pathogens causing wilting/root rot, or both, in recent decades in Mediterranean countries, but also worldwide, with varying percentages of plants presenting symptoms, in some instances up to 60% [52]. Pests and diseases that can affect lavender crops can reduce their longevity from over 10 years to only 3 [6]. Farmers should always look out for emerging pests and diseases and remain vigilant, conducting prompt interventions to prevent the spreading of either old or new pests and pathogens, thereby saving their crops. 

The pests and diseases reported for lavender crops are as follows. 

(1) Fungi *Septoria lavandulae* Desm. causing leaf spot [34], *Rosellinia necatrix* Berl. ex Prill. [6] and *Armillaria mellea* (Vahl) P. Kumm. causing root rot [6,34], *Phoma lavandulae* Gabotto and *Phomopsis lavandulae* causing foliage discoloration and wilting [53] and *Botrytis cinerea* Pers. causing gray mold, and other opportunistic soil-inhabiting pathogens, such as *Fusarium* sp., *Verticillium* sp., *Sclerotium bataticola* Taubenh and *Sclerotinia sclerotiorum* (Lib.) de Bary [52].

(2) Chromists such as *Phytophthora* sp. [53] causing stunted growth, yellowing of leaves followed by wilting and defoliation of lavender. The following species were identified on lavender plants worldwide: *Phytophthora nicotianae* var. *parasitica* (Dastur) G.M. Waterh., *P. cinnamomi* Rands, *P. palmivora* (E.J. Butler) E.J. Butler, *Phytophthora* × *pelgrandis* W.F. Gerlach, Nirenberg & Gräfenhan and *P. cryptogea* Pethybr. & Laff. [52].

(3) Bacterial infections with *Xanthomonas campestris* (Pammel) Dowson [52] and *X. hortorum* Vauterin et al. affect foliage [54], and *Pseudomonas syringae* Van Hall causes wilting of young shoots and occurs in wet weather [34]. Yellow decline is caused by a mycoplasma infection that reduces the life of the lavender crop by a few years, and is more widespread in France [34]; ‘*Candidatus Phytoplasma solani*’ Quaglino et al., is the causal agent of stolbur in lavender crops [55,56].

(4) Nematode *Meloidogyne* sp. is the causal agent of root knot disease [34,57]. 

(5) Arthropods, such as *Thomasiniana lavandulae* Barnes, whose larvae feed under the bark damaging the tops of branches, represent some of the most important pests in lavender; other pests, such as *Hyalesthes obsoletus* Signoret, *Cechenotettix martini* Lethierry, *Eucarazza elegans* Ferrari, *Arima marginata* Fabricius, *Chrysolina americana* L., *Meligethes subfumatus* Ganglbauer, *Argyrotaenia pulchellana* Haworth and *Pterophorus spicidactyla* Chrétien are of varying importance [6]; other arthropods reported are the foam cicada—*Philaenus spumarius* L. causing deformation of leaves and stems [34] and *Sophronia humerella* Denis & Schiffermüller, the lavender moth [53]; 

(6) Alfalfa mosaic virus (AMV) causes bright yellow marks on leaves and debilitates the plants [34,52]. 

A study showed that *L. angustifolia* is more susceptible to stolbur disease (*Phytoplasma*) than cultivated interspecific lavender hybrids [58], and therefore farmers must monitor with care the phytosanitary status of their lavender crops. A recent study suggested that *Salvia sclarea* L. is a host for *Hyalesthes obsoletus*, a vector of *Phytoplasma,* the causal agent of severe decline in young lavender crops; this study, from France, further suggested that species such as *S. sclarea* might act as reservoirs for the disease [55]. A report from Bulgaria indicates that *Philaenus spumarius* L. and *Lepyronia coleoptrata* L. act as vectors of this disease [56]. Such recent reports bring to attention the complex interrelationships at play between disease occurrence and the natural environment. A recent report from China relates the first encounter of *Epicoccum sorghinum* (Sacc.) Aveskamp, Gruyter & Verkley infection of lavender plants causing blackleg disease, with high percentages of up to 50% incidence in lavender plantations in Anhui Province. The symptoms were progressive wilting and necrosis, starting from stems and eventually leading to plant death [59]. 

A study screening lavender crops from Bulgaria reports that healthy crops free of pests can be ensured by having a good agri-background for the crop. Thus, crops located on ventilated slopes with adequate air currents and water drainage and good sunshine exposure are key for maintaining a pest-free crop. In addition, adequate agrotechnical measures implemented during cultivation are also contributing factors for a healthy crop [53]. In this regard, removing diseased plants before pathogen spread is a good approach. In a well-maintained crop located in an adequate position where the ecological factors are optimal for this species, application of chemical control treatments should not be required. Moreover, an investigation suggested that the intensive visiting of the lavender crop by honey bees (*Apis mellifera* L.) effected a protective zone against other pathogenic entomofauna, ensuring a pest free crop [60]. Since this is a melliferous plant, it is preferable that insecticides be avoided. In recent years, there has also been increased awareness of the ecosystem services that agroecosystems biodiversity can provide. Strong evidence from Europe on functional biodiversity and its interplay with beneficial agronomic outcomes offers attractive perspectives that can no longer be ignored [61]. In this sense, encouraging diversity that shelters and protects the natural enemies of pests and encourages pollinators is viable for lavender crops as well. 

#### 3.4.3. The Key to Pruning Lavender

Pruning revitalizes and stimulates the flowering of lavender plants. Lavenders that are pruned often live longer, and pruning is routine in lavender farms [34]. To stimulate flowering, pruning can be conducted in spring (after the danger of frost has past) by cutting the stems to one third. In the summer at harvesting, the flowering tops (inflorescences) are trimmed. Light pruning can be applied in autumn, a few weeks before the danger of frost, by trimming the shoots about 2 cm above the wood. Mature plants can withstand hard pruning. This is applied in old plantations by cutting the plants closer to the ground to stimulate crop rejuvenation [5]. 

#### 3.4.4. Fertilization 

Lavender can survive without any fertilization, but fertilized plants are healthier and more productive. With every harvest and pruning, some of the nutrients that plants extract from the soil are removed, and therefore in time, the capacity of the soil to replenish what has been removed by harvesting slowly decreases [34]. For a healthy crop, lavender plants particularly need nitrogen, phosphorus, zinc, boron and magnesium [31]. Fertilizer is recommended at crop establishment, when 30–50 tons/ha of manure should be incorporated in the soil [5]. The use of phosphorus-based fertilizers has been known to result in almost twice the amount of inflorescence per plant [31]. 

In conventional cultivation technology, chemical fertilization has been shown as beneficial for the lavender crop. Fertilizers with phosphorus and potassium can be applied in autumn [31] in quantities of 70–80 kg/ha P_2_O_5_ and 40–60/ha K_2_O [5], while those based on nitrogen in spring after snowmelt [31] can be applied in quantities of 60–80 kg/ha [5]. 

At the European level, the Committee on Herbal Medicinal Products advises in their ‘Guideline on Good Agricultural and Collection Practice for Starting Materials of Herbal Origin’ (GACP) that fertilizing agents should be applied sparingly, while the substances for growth and crop protection should be either avoided or kept to the minimum [62]. This is understandable given that when buying products for phytomedicine or for their other health-promoting attributes, customers expect a product as natural as possible [11]. In addition, there are environmental concerns about nutrients leaching into natural ecosystems from chemical fertilizers and the corresponding negative consequences, such as the eutrophication of water bodies [63]. Therefore, more environmentally friendly approaches should be considered. Among the green alternatives are the use of organic fertilizers and the leveraging of natural mechanisms to enhance their agronomic traits. 

One strategy with a beneficial outcome, as demonstrated in conditions from south-east Romania [64], is to make use of chemical fertilizers only at crop establishment, and then to transition to organic ones. Because chemical fertilizers release nutrients quickly, they can act as crop starters to stimulate good vegetative growth during the establishment of the plants, and therefore would be best used in the first year of the crop, when there is usually no harvesting conducted. In the subsequent years, the use of slow-release organic fertilizers is more beneficial. In this regard, some authors recommend fertilization in spring with a slow-release product, such as blood, bone or organic manure for the lavender crop [34]. Regarding the type of organic fertilizer used, research has produced evidence of the influence of cow manure, vermicompost and the combination of both on several agronomic traits, such as biomass yield and essential oil quantity and quality. The cow manure application increased essential oil yield by 70% compared to the control, while the combination increased the leaf N concentration by 43%. The main changes in essential oil composition due to different organic fertilization were related to oxygenated monoterpene content, followed by borneol content and camphor content [65].

Among the natural agents that can enhance biomass and modulate secondary metabolites in favorable ways for aromatic plants are soil microorganisms, such as arbuscular mycorrhiza. These fungi enhance plant nutrition, particularly in terms of phosphorus, a nutrient with a crucial role in the biosynthesis of secondary metabolites [66]. In addition, N and some micro-nutrient uptake (Zn, S, Cu, Fe and Mn) by the plant can also be improved through this symbiosis. Studies show that arbuscular mycorrhiza inoculation can increase lavender growth and photosynthetic pigments [67] and significantly increase linalyl acetate content in lavender essential oil [68]. Because mycorrhizal colonization level was shown to be significantly influenced by lavender plant health status, and could be modified by agricultural practices [69], there are more studies needed to elucidate the stability of symbiosis in order to identify ways to assist lavender crops in benefiting from these microorganisms. As an environmentally friendly approach, this should be pursued further. 

### 3.5. Harvest, Drying and Yield

The lavender harvesting starts in the second year of the crop at flowering, which takes place in June–July and continues yearly for 12–15 years in conditions of high productivity. Beyond this time interval, the crop becomes less feasible [5]. The crop enters full productive capacity in the second and third years post-establishment [70]. 

The period and duration of flowering varies with geographical region. In Eastern Europe silvosteppe areas, the flowering, with a flowering duration of 15–20 days, occurs earlier than in colder temperate regions, where flowering can occur later and lasts about 25–30 days [5]. 

The harvested plant part for essential oil production are flowering tops/inflorescence, collected during full flowering [26,28,71]. More specifically, according to different authors, for the highest essential oil yield, the flower harvest should be performed at 50% flowering [5], 60% flowering [26], 75% flowering [31] or towards the end of flowering [72]. For dry flowers, the harvesting is performed when the first flowers start to open, while for seeds, the inflorescence is harvested at the fruiting stage [5]. Harvesting should be performed preferably after 10 a.m. until noon [5], or after midday, according to other authors [73]. However, other aerial plant parts also can be used, such as shoots and leaves with or without stems, but the essential oil yield is lower and the qualitative parameters are also lower [24]. 

In Romania, the flowering shoot yield is 2–3 tons/ha in the first 3 years of the crop, and 5–6 t/ha thereafter [5]. In the climate of the Mediterranean region, the region of origin for this species, the flowering shoot yield varies between 5.5–15 tones/ha [70]. The yield of dry flowers is about 1000 kg/ha. For seed crops, about 300–400 kg/ha of lavender seeds can be obtained [5]. 

The inflorescence is dried in special designate spaces in shade, or artificially at 35 °C. The drying yield is 5–7:1 [5]. A study from Poland showed that the number of compounds in the essential oil from air-dried lavender was higher (60–70 compounds) compared to oven-dried lavender (56–57 compounds), while an essential oil richer in camphor was extracted from oven-dried material [35]. 

### 3.6. Extraction 

Essential oil has been extracted conventionally for commercial purposes by hydrodistillation and steam distillation, with a wide variation for time needed for extraction [5,24]. Generally, the term ‘essential oil’ is used for a plant-derived product obtained by distillation, and the term ‘extract’ is used when other methods are employed [74]. Other methods can also be used for isolating the rich volatile aromatic mixture from aromatic plants, including effleurage, maceration and solvent extraction, to name a few of the more common approaches [2]. Terpene-free lavender oil is obtained by careful vacuum distillation. By this method, it is sufficient to ‘top off’ 10% of the oil in order to make it softer and mellower. It is more soluble in dilute alcohol and has increased stability; therefore it is used in foods [6]

Fresh lavender flowers contain about 0.7–1.4% volatile oil. When the plant material is dried, some volatile oil is lost. One ton of fresh inflorescence results, on average, in about 10 kg of volatile oil. [5]. According to European Pharmacopoeia [75], the content of essential oil should be ≥13 mL/kg of flowers. A recent study re-confirmed that calyx is the main plant part - which accumulates volatile oil in lavender. While calyx alone showed a yield of 1.3%, followed by corolla (0.1%) and leaf (0.05%), the entire flowering top had a yield of 0.7% [76]. It has been known for some time that essential oil yield is 4–5 times higher from lavender flowers than from leaves [24]. 

Extraction methods also influence the essential oil obtained as well as the yield [24]. A comparative study showed that through the conventional hydrodistillation method, the essential oil slowly permeates through membranes and cuticles with some drops of oil remaining stuck on the outside of glandular trichomes or on the plant material, while with the application of microwave-assisted extraction, the glandular trichomes burst open and collapse, releasing the essential oil without residual oil remaining on the plant material [28]. Thermal or hydrolytic degradation and loss of volatile compounds that change the chemical profile can be encountered with different methods used. For example, linalyl acetate partly degrades when hydrodistillation is used, resulting in higher linalool content compared to steam distillation [24]. Comparison between three extraction methods (hydrodistillation, microwave-generated hydrodistillation and microwave hydrodiffusion and gravity), showed the number of compounds in lavender essential oil was similar, but there were differences in their percentage. Thus, essential oil extracted by hydrodiffusion and gravity was richer in oxygenated monoterpenes and particularly borneol. The essential oil extracted by classic hydrodistillation and microwave-generated hydrodistillation was richer in coumarin [77]. 

The optimization of extraction method should be further researched with the purpose of providing guidelines for the processing industry that could ensure minimization of losses and increased efficiency of extraction. However, given that quality exigence is related to price and can also impact further utilization and products obtained, the influence of extraction method on qualitative parameters should not be exceeded by the importance of the attention given to extraction efficiency alone. 

## 4. Genotypes Cultivated and Breeding Trends 

The breeding of MAPs began later than for traditional crops. Investigating germplasm resources is the starting point for breeders [78], and represents the basis for acquiring superior varieties [79,80]. It is necessary to actively develop breeding processes to produce new, highly productive varieties of plants resistant to biotic and abiotic stressors that behave well in culture and are able to ensure a stable plant raw material supply [81]. 

There have been breeding efforts to create exceptional lavenders. *L. angustifolia* ssp. *angustifolia* was domesticated when wild populations were selected for the yield and quality of their essential oil to be cultivated in fields [25]. Since 1600, lavender cultivars have increased in number, and there are instances when the same genotype is found and registered under different names. The oldest one according to some sources is the cultivar ‘Alba’ [7].

**Figure 2:**
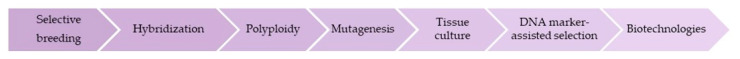
Methods used in developing new lavender genotypes (original flow-chart by Andreea O.).

The modern lavender breeding programs have their beginning in France, and were established later also in Eastern Europe [23], United Kingdom and then worldwide [7]. At first, lavender breeding programs were based on testing, selection and multiplication of the best-performing plants. Then, the breeders conducted hybridization programs between different varieties to explore the effect of heterosis [82]. In Eastern Europe, some locally adapted lavender cultivars were obtained by using a polycross hybridization scheme [83]. Other methods employed by breeders include experimental polyploidy and mutagenesis. Nowadays, targeted breeding is increasingly necessary to optimize lavender improvement programs, and this is now possible due to lavender genome sequencing [23]. Breeding methods used to create new genotypes of lavender are presented in Figure 2. 

Despite its significant importance, the intraspecific diversity of wild, non-cultivated lavender plants is still not fully comprehended [25]. Breeding strategies can be made easier with understanding of phylogenetic relationships and cytogenetic traits [84]. More studies are also required to understand the genetic and environmental factors that result in the production of the lavender chemical biodiversity [24]. Chemotypes are chemically distinct entities within the same species that display genetically inherited chemical signatures, and are important in breeding highly performant cultivars of lavender [25]. 

Andrews and later McNaughton, cited by Lis-Balchin [7], listed more than two decades ago a large number of cultivars alongside their descriptors. Different lavender cultivars have been bred for certain purposes and uses [85]. 

There are some old cultivars that are widely known and still cultivated, such as ‘Munstead’ and ‘Hidcote’, two cultivars bred before 1950 in United Kingdom [7]. Currently, the French cultivar ‘Maillette’ remains widely cultivated across Europe, while in Eastern Europe, the Bulgarian cultivar ‘Sevtopolis’ is widespread, both being used for essential oil production. 

There are many cultivars mentioned in the literature across various geographical regions (Table 1).

A study conducted on 14 wild lavender populations in their native range (France, Italy and Spain), reported 63 volatile organic compounds in the investigated lavender plants across sites, with 10–55 compounds occurring per population of plants [25]. Another study identified 104 compounds in essential oil across 9 native lavender populations from the Western Italian Alps, with the number of compounds per population varying been 63–83 [22]. Wild lavender can still play an important role in breeding lavenders with complex volatile profiles. 

A recent study from Ukraine identified no less than 70 compounds in the essential oil of *L. angustifolia* across seven genotypes, with numbers varying between 39–47 compounds per cultivar [96]. A study from Hungary reported up to 36 compounds in the essential oil from the cultivars ‘Budakalászi’ and ‘Maillette’ [72]. A study from Romania reported 38 compounds in the essential oil from the cultivar ‘Sevtopolis’ [71]. Using cultivars that perform best in certain climatic conditions is vital for successful crops. Therefore, adaptability to local conditions is a trait to be strongly considered in the breeding programs. 

Breeding lavender often requires the sexual reproduction of the plants in order to allow hybridization between valuable parent progenitors. An example of the successful application of hybridization in lavender is the variety ‘Etherio’ which demonstrated an increase in essential oil yield of 78.8% in branches and inflorescences in comparison to the native plants from which it was created. Additionally, the final essential oil had higher quality due to higher concentrations of linalool and linalyl acetate, while eucalyptol and camphor percentages were lower [82].

Traditional plant breeding aims to improve the desired traits in crops, but the process takes place over long periods of time, sometimes exceeding 10 years. For this reason, it is imperative to turn to techniques that allow a faster improvement of the desired characteristics. Ploidy (chromosome number modification) is such an approach [78,101]. Polyploidy occurs naturally, but it can also be caused by factors that inhibit the mitotic spindle formation during cell division (e.g., colchicine). The numbers of chromosomes in *L. angustifolia* differ significantly, ranging from 34 to 75, possibly due to previous genome polyploidization [101,102]. Genome size and the number of chromosomes should be considered when inducing polyploidy in lavenders within the improvement programs [101]. 

More recently, research has concentrated on using metabolic engineering and other molecular approaches to improve oil yield and quality in these plants [4]. It is well-understood that the establishment of an efficient tissue culture system is the foundation for genetic manipulation of aromatic and medicinal plants, and it is desirable to optimize tissue culture protocols for lavender, including callus formation, anther and protoplast culture systems [78]. 

Through molecular hybridization, polymerase chain reaction, and high-throughput sequencing technology, DNA marker-assisted selection, which is based on DNA polymorphism, can be used to identify DNA fragments as markers that are connected to desirable phenotypes, such as high yield, better quality and improved resistance, and can aid in the breeding of new varieties [103]. Zagorcheva et al. [104] highlighted some uses of sequence-related amplified polymorphism (SRAP) markers in the breeding and cultivation of lavender by characterization of the lavender genetic resource pool, the characterization of populations that segregate within the lavender species, genetic mapping studies and marker-assisted selection.

The biochemistry of essential oils and the mechanisms involved in their synthesis, but also the complex genetics of lavender, became available with advancements in genomics and post-genomics [78]. Malli and his research team [102] generated the first draft genome for *L. angustifolia*. It appears that lavender presents a moderately repeated genome of 870 million base pairs (Mbp), which consists of 688 Mbp of non-gap sequences incorporating 62,141 protein-coding genes and 2003 RNA-coding genes. This has great implications in identifying genes that are involved in the biological processes, but also in finding genetic markers that can be used in lavender breeding programs [102]. The role of gene duplication in lavender adaptation is now better-understood, thanks to genetic data from the lavender genome [105]. It also seems that due to the high percent of genes that are duplicated in *L. angustifolia* genome, the species is enhanced for the production of essential oils [102]. Furthermore, identifying genome and molecular foundations of salinity tolerance could assist in enhancing and enabling lavender to make use of and restore salinized unproductive land, or tolerate watering with sea/salty water. Preliminary research for identifying salinity responsive genes in *L. angustifolia* through cDNA amplified fragment length polymorphism explored this potential [106], and calls for further study due to high applicative prospects. 

As a new approach, cisgenics, which involves the introduction of wild strains to local varieties, can be used in lavender breeding [78]. Breeders may also identify the desired gene or characteristic in other organisms and utilize transformation technology to introduce it into new variations, if it is absent from any parental material or closely related species or subspecies. However, there have been far fewer investigations into the transgenic breeding of medicinal plants than into other crop plants due to some limiting characteristics, such as unclear genetic background, high heterozygosity, and numerous sequence repeats [103]. 

A high level of genetic variety within the resource pool is a requirement for starting a long-term breeding program for lavender [104]. Traditional techniques of individual and individual-family selection, polyploidy, chemical mutagenesis and a combination of these techniques are still promising in the breeding of medicinal and essential oil crops [81]. Nevertheless, to develop new types of therapeutic plants, traditional breeding techniques must be combined with molecular biology and biotechnology. The development of contemporary biotechnologies offers beneficial opportunities and new tools for research into breeding medicinal plants [79]. The major objective in lavender breeding is to raise the output of medicinal plant raw material as well as the concentration of a few specific secondary metabolites, especially the content of linalyl acetate. However, special attention must be paid not only to the quality and the quantity of production, but to the genotype stability. Because people expect a natural product, the use of resistant genotypes that do not require pest and disease control treatments are preferred [62]. Due to this, resistance to pest and diseases remains at the heart of breeding efforts, alongside quantitative and qualitative yield enhancement. 

## 5. Overview on the Importance and Emerging Uses of *L. angustifolia*

Alongside traditional uses, there have emerged in recent years many novel ones. Some may currently occupy only a niche, but could increase in importance in the decades to come. Particularly attractive are secondary uses, which enable lavender growers or the processing industry to make the best use of crops, raw materials and by-products. Growers could explore secondary utilization options to increase or supplement their incomes. In addition, industry actors could employ novel uses to monetize the resulting by-products, which would be in line with the circular economy concept of ‘zero waste’.

Due to the increasing number of uses of lavender and the ongoing search to optimize both their cultivation and their applications, choosing the right genotype for target purposes leads to best outcomes. Some cultivars with recommended uses are presented in Table 2.

Many cultivars have been created for essential oil production. Literature indicates that although ‘Maillette’ remains the French standard for the essential oil quality obtained, there are a few cultivars that come close to it: ‘Compacta’, ‘Irene Doyle’ and ‘Twickel Purple’ [85]. Others have been found to be particularly suitable for certain uses: the cultivar ‘Buena Vista’ has been indicated as suitable for pot-pourri, and ‘Blue Mountain’ for hedges. Some cultivars created are highly ornamental and display specific aesthetic characteristics, such as white flowers (‘Celestial Star’), soft lavender-pink tones (‘Hidcote Pink’), vibrant violet (‘Budakalászi’) or dark flowers (‘Hidcote Blue’) [7]. Lavender pallets of colors are well-documented to inspire horticulturists in finding the best ones for their needs. In addition, there are indications for those best adapted to certain conditions, such as those cultivars able to withstand cold winters, like ‘Maillette’, ‘Royal Velvet’, ‘Imperial Gem’, ‘Folgate’ and ‘Buena Vista’ [39]. 

Due to superior volatile profile, *L. angustifolia* is used preponderantly in perfumery and cosmetic industries, while other species of the genus with harsher volatile profiles that are richer in camphor find non-perfumery destinations of use [21,108]. While choosing the right cultivar for the products intended to be obtained is important for lavender famers in order to best monetize their raw material production, recent literature suggests that farmers could also consider novel utilization options for their crops. This could be approached both for a complementary income, or if proven feasible, by transitioning towards more highly advantageous destination of use. However, traditional approaches for the utilization of lavender crops, such as essential oil production, remain the market backbone due to the high demand.

**Figure 3:**
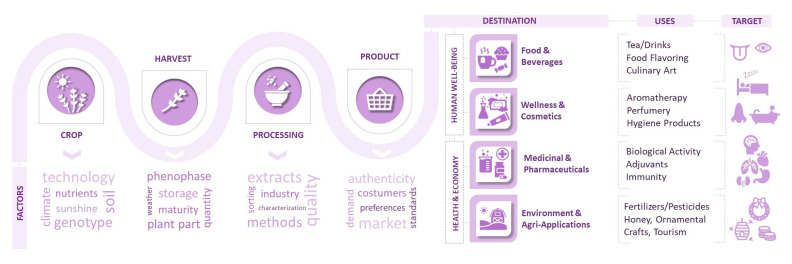
Lavender from crop to products with an overview on the main uses as they are related to human senses, activities and favorable outcomes (original infographic by Ioana C.).

The trajectory of lavender from crop to products is summarized in Figure 3.

### 5.1. Food and Beverages

This category of uses is both new and old. The use of lavender tea is old, but there are more recent uses. In food industry and cuisine, lavender buds or essential oils can be used as flavoring agents. For this purpose, English origin is preferred because has a milder and more pleasant taste. The following food products can be flavored with lavender: candies, chewing gum, condiment blends (chutney, salsa), cooking spices, confectionary, jams and jellies and beverages, such as tea blends, hot chocolate, coffee, lemonade and cocktails. Creative kitchen uses suggested by cookbooks involve the use of lavender for artisanal pizza, ice-cream and home-cooked chicken recipes. Its antimicrobial properties can be employed to prevent food spoilage or incorporated in packages. Lavender essential oil has been tested for meat preservation, but also against a wide range of food-borne pathogens in general [109]. It has been proposed that phenolic extracts from solid waste resulting as a by-product of essential oils extraction could find wide uses in the food industry due to their antimicrobial and antioxidant properties. These can range from food additives to packaging [110]. Lavender-processing by-products rich in polyphenols could be used in the baking industry. Bread with the addition of 2.5% lavender by-product showed higher loaf volume and increased shelf life and consumer acceptability [111]. Active food packaging could be developed using lavender essential oil. Films of starch fulcellaran and gelatin loaded with lavender essential oil at concentrations of 2–6% showed antioxidant and antimicrobial activity, while some physical properties of the films were also enhanced [112]. 

### 5.2. Wellness and Cosmetics

This category of uses is an important one from the economic point of view [4]. Driven by the desire for a healthier lifestyle, the use of natural ingredients in cosmetics is in high market demand. 

Fragrant lavender oil and flowers are used in aromatherapy, detergents, massage oils, perfumes and personal hygiene products, such as shampoo and soap [113]. Aromatherapy is considered effective due to the psychological effects of fragrance as well as the physiological effects of the compounds, which act through limbic system (amygdala and hippocampus) [21]. For aromatherapy, the essential oil is administered through vaporization or direct application on the body in small quantities [109]. Out of the all cultivated lavender species, *L. angustifolia* is the one preferred for perfumery as well as cosmetics [21]. A recent study shows that the biomass resulting as a by-product of essential oil extraction could be used as a source of polysaccharides and incorporated in cosmetic products [114]. Steam distillation produces hydrolats as a by-product, which have a volatile organic compound content between 9–97 mg/100 mL and shows low antimicrobial activity [115]. Although it lacks the linalyl acetate found in essential oil, it presents linalool, α-terpineol and coumarin, among other constituents [116]. This lavender hydrolat presents a floral–herbal scent and is commonly known as ‘lavender water’ and sold for personal and home use. The possible utilization options for lavender water have been insufficiently explored, and since it is a by-product obtained in abundance, new uses could be highly attractive for the processing sector. 

### 5.3. Medicinal Uses and Pharmaceutical Potential

This category of uses has significant potential for the development of novel products in the years to come, as the knowledge of their properties and effects is expanding. 

Traditional medicinal uses for lavender include sleeping aids based on the calming effect of lavender tea and oils, as well as treatments for wounds, lice infestation, migraines, panic attacks and some heart problems, cold, bites, cramps and congestion [109]. Some traditional uses based on previous empirical knowledge were later confirmed with various degrees of confidence through scientific methods [21]. Pharmacotherapy screening on the properties of lavender found indications for their use in relation to anxiety, with strong evidence. Several other indications of use, although with only moderate evidence, were hypnotic/sleep aid, mitigation of perineal discomfort following childbirth, spasmolytic and antibiotic treatments and cancer treatment based on phase I human trials (lavender constituent perillyl alcohol) [113]. Several studies on cell lines revealed cytotoxic effects on cancer cells of specific lavender compounds or essential oils: breast cancer cells, leukemia cells, melanoma cells [117], colon cancer cells and ovary cancer cells [71]. 

Aside from the actions on the central nervous system, lavender was shown to have an antispasmodic effect on both uterine and intestinal smooth muscle, ergogenic effects in sports training [21] and benefits for the circulatory system [118]. 

There is evidence for the antioxidant and anti-inflammatory effects of lavender essential oil [4]. Antimicrobial activity was documented for some common pathogens, such as: *Staphylococcus aureus* Rosenbach, *Streptococcus pyogenes* Rosenbach, *Escherichia coli* Mig., *Enterococcus faecalis* (Andrewes and Horder) Schleifer and Kilpper-Bälz [21], *Candida albicans* (C.-P. Robin) Berkhout, *Pseudomonas aeruginosa* (Schröter) Migula, *Bacillus subtilis* (Ehrenberg) Cohn, *Listeria monocytogenes* (E. Murray et al.) Pirie, *Salmonella* sp., *Enterobacter* sp., *Klebsiella* sp., etc. Results indicated that lavender oil might be useful as a prophylactic or for topical application in surface infections but not for deep-seated infections, with greater effectiveness against Gram-positive than against Gram-negative bacteria [118].

Despite promising results obtained regarding biologic activity, there are some hindrances in making the next step towards developing therapies. One of them is the lack of standardization for the dosages and experimental set-up from scientific literature, which makes it difficult to reach a consensus regarding optimal concentrations. 

In pharmaceutics, essential oils are included in different dosage forms, such as capsules, creams, syrups, suppositories, ointments and aerosols. In the medical field, there is potential for the use of lavender essential oil as a disinfectant for equipment and medical surfaces, or as aerosols in waiting rooms or operating blocks to limit air-borne contamination [109].

One study shows that through fermentative or enzymatic processes involving microorganisms (especially filamentous fungi), the solid by-product fraction resulting from essential oil extraction could be used in the production of various bio-active compounds, such as antimicrobials, antioxidants and various bioproducts with either cosmetic and pharmaceutical activities, opening up novel possibilities for white biotechnology applications [119]. 

Proof-of-concepts from literature in regard to the possibilities for using by-products for valuable compounds are highly attractive. However, processing these products requires highly specialized knowledge and equipment. When transitioning towards implementation, some foreseeable challenges might arise. There would be a need for regional centers where these could be sent or collected, considering that sending by-products over large distances might not be financially attractive. The existence of a processing center might also depend on the number of lavender crops and farms in the area. Perhaps the best way would be for centers where the plant material was distillated to be equipped to also immediately further process, pack and store the by-products. Based on positive experimental data, these technologies should become available to be transferred towards market. 

### 5.4. Environment, Agri-Applications and Niche Uses

This category of uses is not currently as important as the ones above, but is certainly a dynamic one and full of potential. Some uses from this category also emerge in response to certain needs from other sectors. A certain degree of flexibility across the crop-to-products chain might confer stability to the volatility of the demand for certain goods. Therefore, one can consider this category of uses as a potentially transitional one, complementary to the purely traditional utilization options at this point in time. However, in the coming years, it could become the norm across the entire chain due to increasing market opportunities. 

The ornamental use of lavender is highly prized by the wide public. In landscaping, lavenders can be used for borders, hedges, rockery, knot gardens or in pots. Lavender can be mixed with other drought-tolerant plants that originate from dry regions of Mediterranean or Central Asia. Some suggestions for companion plants for lavender are: *Achillea millefolium* L., *Arabis* sp., *Armeria maritima* (Mill.) Willd., *Artemisia* sp., *Buxus microphylla* Siebold & Zucc. cv. ‘Winter Gem’, *Centranthus ruber* (L.) DC., *Echinacea purpurea* (L.) Moench, *Gaillardia × grandiflora* hort., *Hypericum olympicum* L., *Kniphofia uvaria* M., *Oenothera* sp., *Penstemon* cv. ‘Firebird’ and *Rudbeckia hirta* L. A highly successful combination is lavender and antique roses, with attention to cultivars, since modern roses do not tolerate drought very well [39]. 

Lavender farms have a first-rate opportunity to use their crops to earn additional income or create goods for complementary income [9]. As it is a melliferous plant, the quantity of honey obtained from lavender is about 50–100 kg/ha [120]. The distinguishable marker of *L. angustifolia* honey is lower phenylacetaldehyde and higher heptanoic acid content [121]. 

Lavender could also be integrated in the strategic marketing of rural tourism destinations to contribute to attracting additional service-based income to sustain rural communities by diversifying income-generating activities in lavender farms. A study from the Isparta province of Turkey, an important cultivation area for lavender and lavandin, revealed that promotion through social media and influencers were important in attracting tourists during the two-month interval in the summer when crops were in bloom [122]. Rural tourism-building around lavender crops and products opens opportunities for partnerships with small businesses to provide locally obtained goods derived from locally sourced lavender that could contribute to the economic stability of the families and communities that depend on lavender. 

Crafts present interesting utilization options that do not require costly equipment and investments, nor highly trained operators. In addition, crafts marketing could be joined with rural tourism to create short value chains, or considered as standalone complementary utilization option. Among the potential uses, such as fresh cut flowers, dried buds and wreaths are the most common [107]. Sachets filled with lavender flowers for their soporific properties to be placed under the pillow [113] or crafts for other home uses could be easily created on the farm and sold locally. 

The environmental application is related to the phytoremediation capacity of lavender on heavy metals contaminated soil. Study shows that lavender is a hyperaccumulator of Pb and an accumulator of Cd and Zn. Although the biomass can be adversely affected by soil pollution, the essential oil obtained can still meet the market standards and is not contaminated [123]. As for their accumulation, this can vary by species. While some lavenders contained higher levels of heavy metals accumulated in their leaves, *L. angustifolia* accumulated them in roots and stems [124]. Lavender could be employed for the use of contaminated soil of otherwise unproductive, polluted land. However, the resulting contaminated biomass poses further environmental issues, such as storage and potential leaching of heavy metals into the environment. The possibilities for using the resulting solid by-products is limited due to contamination. Therefore, secondary utilization options for the resulting contaminated biomass should be sought that will in turn make lavender cultivation on heavy metal contaminated soils more attractive. The influence of heavy metal stress on the quality of essential oil should also be researched. 

It has been proposed that aromatic plants could restore unproductive as well as marginal lands to the status of productive agroecosystems. Given the fact that these species can often thrive in adverse conditions where food crops fail or are less feasible, their cultivation with low input and high output in the form of valuable essential oil is attractive [125,126]. In this regard, *L. angustifolia* is a suitable crop for abandoned and hilly marginal lands. The value of agronomic production per hectare of land is 5800 EUR, according to data from Italy, if it is used for essential oil production [127]. Lavender plants are adversely affected by salinity stress, with both morphometric as well as essential oil yield significantly reduced. The interaction of bio-dynamic preparations and biofertilizers were shown to alleviate salt stress effects in lavender and enhance essential oil yield [128]. These further open up the possibility of extending the cultivation of lavender without competing for fertile agricultural soil. 

Protection of cultural heritage monuments against degradation could be aided by lavender. Lavender essential oil (10–100 μL/mL) was tested against fungi isolated from cultural heritage objects. Sensitive isolates were *Epicoccum nigrum* Link and *Penicillium* sp., proving lavender essential oil might be used to mitigate the activity of some biodeteriogens [129]. 

In agriculture, there are some interesting uses for products and by-products of lavender. The solid by-product resulting from essential oil extraction has been used as soil fertilizer or converted into a fuel source. However, these cheap valuable resources may currently be of industrial interest, and might find better uses [119]. Lavender essential oil has been shown to have a moderate inhibition effect on the growth of plant pathogens, such as *Alternaria* sp., *Botrytis cinerea*, and *Colletotrichum* sp., with the most effective concentration being 1000 μL/L [130]. The loss of stored food production due to pests poses particular challenges that call for sustainable approaches, due to the toxicity of some chemicals used. An experiment demonstrated that lavender essential oil is effective against *Acanthoscelides obtectus* Say [131]. The use of lavender essential oil as insecticide for stored grains, although effective, might not yet be entirely feasible, considering the costs and high quantities needed [109]. 

Because the lavender scent effectively repels moths and flies, for this purpose it is placed in closets and drawers, with proven insecticidal activity [118]. For home use, the required quantities are smaller than for industrial-scale use, and therefore is more widespread. Room fresheners and pot-pourris for home are common uses [3]. Since other species of the genus *Lavandula* are known to have higher camphor content than *L. angustifolia*, they are more suitable for use as insect repellents, as well as more effective antimicrobials [21]. 

In animal farming, there is evidence for the advantages of using lavender for rearing healthy broiler chickens. Chickens provided with water containing 0.4 mL/L lavender essential oil during the second period of rearing (days 22–24) had enhanced body weight, with 6.35% higher than the control. In addition, the treatment improved the gut health of chickens [132]. Dietary lavender extract (1.0–1.5%) administered to common carp (*Cyprinus carpio* L.) was associated with the suppression of crowding stress, inflammation and oxidative conditions, as well as of increased immune responses [133]. Lavender was shown to have acaricidal capacity and could be used against ticks and in a veterinary context [134]. 

Some niche uses with potential emerging importance or experimental value were also proposed. Lavender essential oil was showed to inhibit the growth of some Mediterranean weeds, suggesting its potential use as bioherbicide [135]. Novel cellulosic fiber with a crystallinity index of 65% was obtained from lavender stems [136]. Lavender essential oil was shown to also have an application in the leather tanning industry [137]. 

## 6. Essential Oil Standards for *L. angustifolia*

Due to the increasing market demand as well as the expanding use of essential oils, the development of regulations, guidelines and standards aiming to maintain a high level of quality and safety remains a priority. Exigence is necessary for ensuring that buyers are receiving what they paid for, and in addition, depending on the destination of use and industry, for ensuring the derived product quality is not adversely affected. 

### 6.1. Sensorial Quality of Essential Oil 

The characteristic scent of lavender oil is fresh floral, resembling the flowering tops of the plant [38]. Oxygenated monoterpenes are the bulk constituent of lavender essential oil and responsible for the characteristic scent. The common monoterpenoids in lavender essential oil are: alcohols, esters, ketones and oxides [24]. The main component in lavender essential oil is linalool, both esterified in the form of linalyl acetate as well as free [92].

True lavender essential oil is appreciated due to its softer olfactory bouquet in comparison to that of other species of the genus [7,24]. The essential oil obtained from the flowers has a milder fragrance than the one obtained from leaves or other plant parts. The essential oil from the leaves and stems is higher in 1,8-cineole and camphor, which are responsible for harsher notes [24]. The linalyl acetate content determines the superior or inferior quality of lavender essential oil [92], and this constituent is also responsible for the floral–woody sensory character of the essential oil [24]. A camphor content exceeding 1.2% reduces the aroma quality by giving a fresher accent, while α-terpineol gives the desired lilac-like scent. It has been proposed that terpineol-4-ol in higher concentrations (over 2%) diminishes the essential oil value by giving it a grass-like scent [92]. These aspects related to aromatic quality are important, considering that essential oils are further used in perfume industry. Therefore, a balance between components is a defining sensorial quality. 

### 6.2. Current Standards and Trending Guidelines for Essential Oil Quality 

Lavender essential oil must have the appearance of a clear liquid, pale yellow in color. To meet the standards, at 20 °C, the relative density must be between 0.878% and 0.892%, the refractive index between 1.457 and 1.466 and the optical rotation between 12.5° and 6°. To obtain a clear solution with one volume of authentic lavender essential oil, should not be required to use more than 2–3 volumes of ethanol 70% or 75%. The maximum acid values should be between 1.0–1.2, while ester values should be between 90–160 [38].

The essential oil profile of lavender analyzed by gas chromatography must present characteristic compounds. Representative compounds are presented in Table 3, according to current standards from literature. These are given for clonal and spontaneous lavender, respectively. The latter refers to standards for essential oil obtained from lavender that was obtained exclusively by seed and grew spontaneously or was cultivated in the south of France. Clonal lavender refers to those plants obtained from crops propagated through cuttings [38]. 

One study showed that volatile profiles of both the calyx and whole flowering tops are consistent with the lavender ISO (International Organization for Standardization) standard, but by contrast, the volatile profiles for the corolla and leaf alone are not within lavender standard ranges. Compared to the corolla, leaf and entire flowering top, the essential oil from the calyx is low in camphor and borneol, hence its superior characteristics. If an abundance of leafy plant material is harvested once with the flowering tops, the quality of the essential oil obtained could be unintentionally reduced (compared to carefully harvesting flowering tops alone) and will present elevated camphor levels [76]. 

There are various certification schemes for agri-sector products worldwide that can attest to certain qualitative particularities of raw material and final product, and these can be grouped into several typologies [138]. Among these, some that may present potential interest for lavender are presented below:

- good agricultural practices (GAP)
- origin certification schemes that guarantee specific origin/quality/attributes
- organic product schemes
- multi-purpose schemes that can combine GAP and quality management
- traceability and safety schemes
- other schemes: non-GMO, Fairtrade [138].

Within the European Union, organic essential oils must be obtained from crops that comply with European and national regulations for organic agriculture [139]. The land must be converted and applicants must undergo inspections on site. Examples of some organic lavender essential oil certifications in France are ECO-Cert, Qualité-France, SOCOTEC [6].

In the European Union, the regulation of commercialized essential oils is under the incidence of Regulation EC Number 1907/2006 EC on ‘Registration, Evaluation, Authorization and Restriction of Chemicals’ (REACH) [140], and other regulations, depending on destination of use, for example, when the destination of use is flavorings in the food industry (Regulation EC Number 1334/2008) or products in the cosmetic sector (Regulation EC Number 1223/2009). Through the REACH regulation, the ‘European Chemical Agency’ (ECHA) was instituted with the purpose of gathering, managing and supervising the registration of chemicals by the manufacturers and importers. In addition, two entities, the European Federation of Essential Oils (EFEO) and the International Fragrance Association (IFRA) release guides on substance identification and environmental assessment guidance on essential oils that assist both manufacturers and other industry and market actors [141]. 

In general, variations in the composition of essential oils of aromatic plants can influence their biological activities [17], and it was proposed that the synergistic effect of various compounds within an essential oil contributes to their overall effectiveness [142]. 

### 6.3. Safety and Authenticity Issues

In general, the aroma profile of essential oils can be given by a dominant constituent or a mixture of constituents, depending on botanical identity [17]. Given the high demand in late years, adulteration is not uncommon across supply chains of essential oils [74,143], and consists in the addition of cheaper essential oils and cheap synthetic materials, or dilution with mineral or vegetable oil, among the most common alterations [74,144]. The simplest way to test for adulteration of an essential oil with vegetable oils is to place a drop on the filter paper and examine it after 24 hours. If a translucent spot is observed, then it indicates that fatty acids (e.g., vegetable oils) were added to that essential oil [74]. The adulteration of lavender essential oil is also achieved by the addition of lavandin and/or spike lavender essential oil, which have higher camphor levels (>6%). Therefore, to determine the authenticity and quality of lavender essential oil, it is important to distinguish the cause of elevated camphor levels. This could be attributed to either the addition of lavandin/spike lavender or to mixing lavender flowering tops with high quantities of lavender leaves for extraction [76]. The usual instrumental procedure to differentiate between authentic and inauthentic/adulterated lavender essential oils is to evaluate the chemical composition in regard to the normalized percentage area or true quantitation of the diagnostic markers, which must be then compared against reference thresholds stipulated in pharmacopoeias or ISO norm monographies [74]. Investigation of 72 lavender essential oil samples showed that unidentified/commercial samples exhibited wider variation in their composition than authentic ones of known origin. As for the indicator compounds, in some samples 1,8-cineole, linalool, camphor, and linalyl acetate exceeded the ISO standards, while 3-octanone, *cis*/*trans*-β-ocimenes, lavandulol and terpinen-4-ol were outside the range of the authentic ones. Authors showed that ISO standards alone are not always sufficient to distinguish between authentic and adulterated essential oil. The Q-Index method based on multiple markers was shown as a highly sensitive tool for discriminating between genuine and adulterated volatile oils [145]. As for the borderline samples that cannot be placed clearly in either category, enantiomeric recognition and an absolute quantitative analysis of a set of marker compounds can be used in addition to the normalized relative abundances for identifying adulterations [74]. 

When it comes to essential oils, safety and efficient doses are aspects to be considered in their use and application [142]. Because authentic essential oils are highly concentrated, they require caution when they are being used [146]. However, lavender essential oil is considered one of the mildest known plant-derived essential oils, and therefore has been used for topical application undiluted on skin. One of the few studies on the cytotoxicity of lavender essential oil showed that dermal cells presented a viability of 80%–100% at concentrations up to 0.125% (*v*/*v*), but not beyond [147]. This suggested that dilution of lavender essential oil when it is used on skin is prudent to prevent sensitization. Results also suggested that linalyl acetate might have a higher chance of causing a skin reaction than the linalool from the composition of lavender essential oil [147]. There have been conflicting reports in the scientific literature on the potential estrogen-like or anti-androgen action of lavender essential oil. A recent critical review of scientific literature by Hawkins et al. [148], found no indication based on the current knowledge that there is any link between lavender essential oil and endocrine disruption in children. 

## 7. Summary of the Factors Influencing Essential Oil of *L. angustifolia*

The numerous factors influencing essential oil characteristics in aromatic plants can be divided into two main categories: (1) before and up until harvesting and (2) post-harvesting factors. The first category of factors refers to all those aspects that influence the living plant, and directly reflect on the quality of the biologic material at the time of harvest. The second category refers to all those aspects that intervene post-harvest and involves what happens to the biologic material after it is collected. The two categories are interlinked to an extent, since all together influence the end result. 

The value of herbal medicines and aromatic plants is directly related to their application, which depends on the consistent quantitative and qualitative composition of the biologic material provided. However, due to cultivation and processing there can arise a substantial variability. The goal is to reduce such variability as much as possible and at the same time to increase the level of qualitative uniformity. 

### 7.1. Factors Related to Plant Biology, Cultivation and Harvesting

Factors intrinsic to the plant are some of the most important. Ontogeny influences the accumulation and composition of essential oil and is therefore found in relation to the optimal time of harvest [17,27]. For this reason, specific phenophase must be defined for this operation [5]. During the development of flower and inflorescence of lavender, the content and composition of essential oil varies [92]. Usually, younger aromatic plants synthesize more essential oil, but the older ones have a richer composition [2]. There are variations from one year to another for the same genotype of lavender [92]. A recent study from Italy showed that the linalool content of lavender essential oil presented high positive correlation with the age of the lavender plant with an increasing trend between the first and fourth years, in contrast to the linalool acetate trend. Similar increasing content with age was observed for two other characteristic components of lavender essential oil: 3-octanone and α-terpineol [127]. A study from Hungary showed that only two out of eight cultivars displayed significant differences in the composition of their essential oil with plant age [149]. Plant organs can influence the essential oil composition, as shown by Wilson et al. [76], the highest quality being obtained from flowers; therefore, the inclusion of stems and leaves should be kept at a minimum during harvesting. 

Agronomic factors, such as the genotype used [72,96] and aspects related to the cultivation technology, such as fertilization [64,65] and irrigation [35], were shown to influence the essential oil obtained from lavender. Some factors are very specific, such as the time of day at harvest [5,73]. It was proposed that there is also a relationship between lavender plant pollinators and optimal essential oil production, considering that harvesting is performed towards the end of flowering and the unpollinated flowers drop down early [150]. 

Among the environmental factors, the microclimate, such as temperature and precipitation distribution along some geographical particularities, can contribute to differences among chemotypes in aromatic plants [17]. A study from Hungary showed that weather conditions—particularly precipitation, influenced the linalool to linalyl acetate ratio in lavender [149]. 

There are also geographical factors influencing agronomic traits in the genotypes of lavender. Results showed that an ecological gradient created germplasm heterogeneity. Thus, intra-specific variations in secondary metabolites both qualitatively and quantitatively were associated with the latitudinal gradient [22]. Furthermore, lavender grown at higher altitudes has higher essential oil content [24]. 

### 7.2. Factors Related to Drying, Extraction and Storage

Pre-processing aspects can influence the essential oil obtained, such as drying methods [35]. Storage can also play a role. Research into two varieties of lavender conducted over a period of eight years showed that during long-term storage of dry flowers, the essential oil content decreased with a mean of 2.56% per year, with notable changes in the volatile profile as well [87]. 

Regarding distillation, several parameters, such as temperature, pH, duration of extraction, aqueous medium—all can influence the essential oil obtained [24]. Comparative investigations also showed that the method of extraction can also influence the parameters and efficiency of essential oil extraction from lavender [28,77]. Once obtained, the stability of essential oils obtained is important for maintaining long-term adequate properties and characteristics [10]. In this regard, optimizing the techno-functional delivery systems in the applications related to lavender essential oils (either for cosmetic products and pharmaceutical use) meant to increase their stability and effectiveness are noteworthy research approaches. Research showed that encapsulation within edible bio-polymers enhanced the thermal stability of lavender essential oil, while emulsions of lavender essential oil nano-droplets can result in enhanced shelf life stability [151]. Furthermore, encapsulation of lavender essential oil could ensure superior applications against pathogenic microorganisms [152,153]. 

## 8. Conclusions

Among hundreds of essential oil-bearing plants, *L. angustifolia* remains one of the most valuable. Its increasing market demand is driven by consumer behavior because of its use being associated with a healthy lifestyle, while expanding applications in various industries are monetizing this trend. The quality of the essential oil obtained can be influenced by a variety of factors that occur either before or after harvest, and standards are set in place to define the range of acceptable variability. 

Lavender crops have increased in recent years, but they do not necessarily compete with food crops for agricultural land, because lavender can be grown on marginal, less productive land or on land contaminated with heavy metals, because it is a low-input crop, while the resulting essential oil is free of heavy-metal contaminants. 

The main trends in the cultivation of lavender are related to the use of environmentally friendly approaches for the production of a high-quality raw material, such as organic fertilization and microbial preparations (e.g., arbuscular mycorrhiza fungi). Breeding efforts making use of novel findings on wild genotypes from populations from the natural range could aim at creating cultivars with more complex volatile profiles. In addition, biotechnology has been used in preliminary studies to screen for either tolerance or susceptibility to various factors (e.g., salinity, diseases) that can impact the crop, and these should be further researched. Performant genotypes and optimized technology packages are needed to ensure a steady supply of raw material of high quality. Improved methods of extraction should be strongly considered and extended in use, such as microwave-assisted, hydrodiffusion and gravity extraction. Long-term stability of essential oils could be achieved by their encapsulation, to prolong shelf-life quality as well as enhance techno-functional delivery systems in their applications. After essential oil extraction results some by-products, such as solid biomass and hydrolat, which could find new uses and thus generate new streams of value. 

The greatest challenges come from the increasing incidence of lavender diseases. Worrisome reports exist regarding increasing stolbur incidence caused by ‘*Candidatus Phytoplasma solani*’ in Europe, where most lavender crops are located, as well as first accounts of severe *Epicoccum sorghinum* infection of lavender crops from China, which could become a worldwide issue in a short while. These pathogens can drastically reduce the life of the crop from one decade to just a few years, therefore threatening the supply chain of lavender raw material. In addition, there are some unresolved concerns regarding the adulteration of lavender essential oil on the market that must be addressed. Certification schemes to guarantee authenticity, origin and quality should become mainstream for lavender products. 

## Figures and Tables

**Table 1:** Genotypes of *L. angustifolia* cultivated in various regions according to literature.

Region	Country	Cultivars and Source
Eastern Europe	Bulgaria	‘Hemus’, ‘Yubileyna’, ‘Druzhba’, ‘Sevtopolis’ [86]
	Czechia	‘Krajová’, ‘Beta’ [87]
	Hungary	‘Budakalászi’, ‘Hidcote’, ‘Maillette’, ‘Munstead’ [72]
	Moldova	‘Aroma Unica’ [88]; ‘Moldoveanca 4’, ‘Vis Magic 10’, ‘Alba 7’ [83]
	Poland	‘Hidcote Blue Strain’, ‘Hidcote Blue’ [89]
	Romania	‘De Moara Domnească’ [5]; ‘Codreanca’ [90]; ‘Maillette’, ‘Vera’ [91]; ‘Moldoveanca 4’ [92]; ‘Alba 7’ and ‘Vis Magic 10’ [93]; ’Buena Vista’, ‘Hidcote’ [64]; ‘Provence Blue’ [29]; ‘Sevtopolis’ [71];
	Russia	‘Prima’, ‘Record’, ‘Sineva’ [94]; ‘Voznesenskaya 34’, ‘Rannyaya’, ‘Yuzhanka’, ‘Voznesenskaya Aroma’ [95]
	Ukraine	‘Record’, ‘Sineva Nadii’, ‘Zmijuchka’, ‘Pink Flamingo’, ‘No. 463-20’, ‘No. 1-469’, ‘1-2-20’ [96]
Southern Europe	Greece	‘Etherio’ [82]
	Italy	‘Maillette’ [97]; ‘Rosa’ [98]
Western Europe	France	‘Matheronne’, ‘Maillette’, ‘Diva’, ‘Rapido’ [25]
Northern Europe	United Kingdom	‘Ashdown Forest’, ‘Blue Cushion’, ‘Miss Donnington’, ‘Fring’, ‘Dwarf Blue’, ‘Heacham Blue’, ‘Hidcote’, ‘Imperial Gem’, ‘Loddon Pink’, ‘Loddon Blue’, ‘Munstead’, ‘Nana Alba’, ‘No. 6’, ‘No. 9’, ‘Princess Blue’, ‘Rosea’, ‘Royal Blue’ [7]
Worldwide	Australia	‘Tuulong’, ‘Lavender Lady’ [7]
	China	‘Jingxun 2’ [99]
	Japan	‘Hayasaki’, ‘Youtei’, ‘Hanamoiwa’, ‘Okamurasaki’ [7]
	New Zealand	‘Blue Mountain’, ‘Avice Hill’ [7]
	Turkey	‘Raya’, ‘Munstead’, ‘Vera’, ‘Silver’ [100]
	United States	‘Betty’s Blue’, ‘Buena Vista’, ‘Lisa Marie’, ‘Royal Velvet’, ‘Gray Lady’, ‘Irene Doyle’, ‘Lady’ [7]

**Table 2:** Some *L. angustifolia* cultivars suitable for different uses [107].

Recommended Uses	Cultivars	Characteristics
Essential oil	‘Maillette’	standard in France and ISO
Fresh cut flowers	‘Buena Vista’	rebloomer, long flowering
Crafts	‘Folgate’	retains color when it dries, good for wreaths
Culinary buds	‘Royal Velvet’, ‘Melissa’, ‘Betty’s Blue’	mild/gentle flavor, good for deserts, food and tea
Dried buds	‘Buena Vista’	mild scent; for pot-pourri and sachets
Landscape	‘Munstead’	small compact bush, strong scent, suitable for pots

**Table 3:** Standards for *L. angustifolia* essential oil quality.

Compound	European Pharmacopoeia [75]	ISO 3515:2002 [38]				
		Spontaneous Genotype	Clonally Propagated Lavender			
		France (from Seed)	France ‘Maillette’	Bulgaria	Russia	Other Origins
lavandulyl acetate	>0.2	>2	<1.3	2–5	1–3.5	<8
lavandulol	>0.1	>0.3	<0.5	>0.3	>0.1	<3
3-octanone	0.1–5	traces; <2	1–2.5	0.2–1.6	<0.6	<3
terpinen-4-ol	0.1–8	2–6	0.5–1.5	2–5	1.2–5	<8
linalol	20–45	25–38	30–45	22–34	20–35	20–43
linalyl acetate	25–47	25–45	33–46	30–42	29–44	25–47
limonene	<1	<0.5	<0.3	<0.6	<1	<1
camphor	<1.2	traces; <0.5	<1.2	<0.6	<0.6	<1.5
α-terpineol	<2	<1	0.5–1.5	0.8–2	0.5–2	<2
1,8-cineole	<2.5	<1	<0.5	<2	<2.5	<3
β-phellandrene	n/a	traces; <0.5	<0.2	<0.6	<1	<1
*cis*-β-ocimene	n/a	4–10	<2.5	3–9	3–8	1–10
*trans*-β-ocimene	n/a	1.5–6	<2	2–5	2–5	0.5–6
(S)-linalyl acetate	<1	n/a	n/a	n/a	n/a	n/a
(S)-linalol	<12	n/a	n/a	n/a	n/a	n/a

Note: numeric values in table represent %; minimum—maximum; n/a—unspecified.

## Data Availability

Not applicable.
